# Quality of life of post–stroke patients and their caregivers

**Published:** 2010-08-25

**Authors:** JA Opara, K Jaracz

**Affiliations:** * Academy of Physical Education in KatowicePoland; ** Medical University in Pozna̤Poland

**Keywords:** caregivers, quality of life, rehabilitation, social support, stroke

## Abstract

With improvements in health care, more people survive stroke but many have to cope with the physical, psychological, social and functional sequelae, resulting in increased personal and public costs. Cerebral stroke causes a significant deterioration of the patient's functioning and worsening of her/his quality of life. Long–term disability caused by stroke is a common problem in all countries and its incidence increases markedly with advancing age.

The assessment of the Quality of Life could be as well the evaluator of sequelae of stroke as an indicator of the effectiveness of the post–stroke rehabilitation. In this review article, the contemporary state of art in assessment of the post–stroke Quality of Life has been presented. The emphasis was placed on participation in terms of personal factors and environmental factors of post–stroke persons and their caregivers.

## Quality of Life post stroke measures

Quality of Life (QoL) assessment has been an important part of the evaluation of stroke patients and their treatment for more than 30 years. QoL is difficult to define and no universal definition of this term exists. However, there is a general agreement that QoL is a multi–dimensional construct that consists of at least three broad domains: physical, mental and social. Researchers and physicians have often used the health–related quality of life concept in the field of medicine, which specifically focuses on the impact of an illness and/or the treatment on the patients' perception, of their status of health, and, on subjective well–being or satisfaction with life [1]. The impact of stroke on health related quality of life may be disastrous; stroke can affect multiple domains of life. To assess these consequences several instruments have been developed. Most of them are questionnaires based on a patient's subjective self–report or self–evaluation. Some of these tools provide information about perceived health status, for example: physical and mental functions, ability to perform everyday activities/roles or the limitation in performing these activities/roles. The other scales capture an assessment of well–being or positive/negative evaluation of particular life domains or satisfaction with life (or specific life domains). There are also questionnaires which produce both information about perceived health status and subjective evaluation [2]. The distinction is made between generic and specific measures. The latter involve items concerning a particular disease or health problem and are considered more sensitive than the generic ones, especially when detecting changes or differences among treatments. Ferrans highlights that when choosing an instrument for a particular study, a researcher should be conscious about the type of information that the very instrument elicits. The reason is that the nature of the self–report of the health status and the subjective evaluation of the well–being is different, it is influenced by different factors, and consequently these two types of QoL data correlate with each other only moderately, even if they assess the same QoL domains. Furthermore, it is crucial that the instrument fits with the aim of the study.  Table 1 presents basic practical information about the most widely used QoL measures for post–stroke patients. They all have accepted psychometric properties (validity and reliability), however none of them is ideal [3–15]. 

**Table 1:** Measures of quality of life after stroke

Name of the instrument	Covered domains	Time to complete (minutes)	Generic/specific	Type of information: perceived health status/ evaluation
EuroQol (The EuroQol Group, 1999) [3]	mobility, self–care, usual activities, pain/discomfort, anxiety/depression and an overall evaluation of health	8	generic	perceived health status
McMaster Health Index Questionnaire (Chambers et al., 1976) [4]	physical, emotional, social	20	generic	both
Nottingham Health Profile (Hunt et al., 1981) [5]	pain, physical mobility, emotional reactions, energy, social isolation sleep	5	generic	perceived health status
London Handicap Scale(Harwood et al., 1994) [6]	mobility, physical independence, occupation, social integration, orientation, economic self– sufficiency and an overall handicap severity score	5	generic	perceived health status
Reintegration to Normal Living Index (RNLI) (Wood-Dauphinee and Williams, 1988) [7]	daily functioning daily activity (work and school); recreational and social activities; general coping skills perception of self presentation of self to others	10	generic	perceived health status
Frenchay Activities Index (Holbrook and Skillbeck, 1983) [8]	domestic chores, leisure/work, outdoor activities	5	generic	perceived health status
36–Item Short–Form Health Survey – SF–36 (Ware et al. 1992) [9]	physical functioning, role limitations due to physical health, bodily pain, general health perceptions, vitality, social functioning, role limitations due to emotional problems mental health	10–15	generic	perceived health status
Stroke–Adapted 30–Item Version of the Sickness Impact Profile (SA–SIP 30) (Van Straten et al.,1997, 2000) [10, 11]	body care and movement, social interaction, mobility, communication, emotional behavior, household management, alertness behavior, ambulation	15	specific	perceived health status
WHOQOL – Bref (Skevington et al., 2004) [12]	physical, psychological, psychological, social relationships, environment, and general satisfaction with life and health.	10–15	generic	both
Ferrans and Powers Quality of Life Index (stroke version) (Ferrans and Powers, 1985) [13]	health and functioning, social and economic, psychological/spiritual, family	20–30	specific	evaluation
Stroke Impact Scale (SIS–59) (Duncan et al., 1999) [14]	strength, hand function, activities of daily living, instrumental activities of daily living, mobility, communication,emotion, memory and thinking, participation	15–20	specific	perceived health status
Stroke Specific Quality of Life Scale (SS–QOL) (Williams et al., 1999) [15]	mobility, energy, upper extremity, function, work/productivity, mood, self–care, social roles, family roles, vision, language, thinking, personality	10–15	specific	perceived health status

## Burden and quality of life in caregivers of stroke patients

Stroke has a great impact not only on the patients' lives but also on the lives of their caregivers. The Carers of stroke patients provide informal care ranging from physical help to psychosocial support. As a result, these carers may experience high levels of burden, associated with characteristics of the patients and of the carers themselves. This burden can result in a deterioration of the carers' health status, social life and well–being. About 80% of stroke patients return home after the acute hospitalization and at least one-half of them require permanent or temporary help from other people in the home setting. This help is usually provided by the closest family member often a spouse or a child, most frequently a daughter who lives with the patient. Family caregivers provide basic personal care, help the patients to perform daily activities, give emotional support, and organize medical and social community service [16]. 

Caring for stroke patients is burdensome and may influence several objective and subjective aspects of the caregiver's life, such as physical and emotional health, morale, work life, finances, social mobility, interpersonal relationships and sex life. It has been reported that an elevated level of anxiety and depression was present in 17% to over 50% of subjects in the studies on the psychological consequences of caregiving, and, in most of these studies, it was higher than both available norms or comparison of control groups [17–20].

The multi–faceted impact of caregiving has been established in several studies carried out since 1988 when the first articles on burden of stroke carers were published [21–23]. Ross and Morris (1988), one of the pioneer researchers in the study of burden, who assessed 20 co–resident spouses of aphasic stroke patients, concluded that the degree of perceived strain had been substantial and comparable to spouses of people with dementia [24]. This was confirmed many years later by Draper et al. (1992) and by Thommessen et al. (2002) who examined family carers of the elderly with stroke, dementia and Parkinson's disease [25, 26]. The obtained results showed that the caregivers perceived a similar type and level of psychosocial burden and psychological morbidity, independent of the disease.

The elevated burden among stroke victim carers seems to be long lasting. The existing studies, mostly cross–sectional, show that high level of burden is experienced in 25% of close family members at 1 month after stroke, in 28% at 2 months, in 28% – 39% at 3 months, in 31% – 40% at 4 – 6 months, and in 51% at 1 year after stroke. [20, 22, 27–29]. More longitudinal studies are needed to evaluate the evolution of the burden over time. So far, only few researches have documented its changes prospectively. Vincent et al. noticed a decrease in burden between the 18th – 24th day and 6 months in 197 carers of people who had a stroke. Only one domain of burden remained stable, namely ‘caregiver social life’ [16]. Similar results were documented by McCullagh et al. (2005) in 232 caregivers of stroke victims between 3 months and 1 year after stroke [30]. Moreover, Visser – Meily et al. noticed a decrease in burden between 1 and 3 years in 23% of 119 carers, however the perceived burden did not change in 60% and worsened in 17% [20].

The severity of burden perceived by carers depends on a variety of factors, both from the caregivers' and the patients' side. Many of them were listed in a recently published paper by Vincent et al. Additional searching literature confirmed Vincent's findings and revealed a few more significant correlates or predictors of the burden [16]. 

They may be summarized as it follows: (1) factors from a patient's side: low functional status [27, 30],  the presence of depression [30, 31], the presence of behavioral and cognitive disturbances [25, 26, 32], being a male [25, 33], older age [16], and the presence of comorbidities [34]; (2) factors from a caregiver's side: older age [32], being a female [16], not being employed [16], being the care receipment's daughter– in– law [34], the amount of surveillance time [29, 30], the presence of depression [19, 35], the presence of disability [36], sense of coherence [35] and non informal social support of the caregiver [28, 32, 37]. 

The burden and strain have been the dominant paradigm in assessing the impact of stroke on the caregivers' life. These studies however capture only the negative consequences of the caregiving role. It is worth highlighting that the caregivers not only perceive burden, but also may experience positive emotions such as satisfaction, pride, gratification and feeling closer to their partners [17, 25, 36]. This is one of the reasons why the QoL concept, which seems to be broader than burden, is also important. QoL reflects well–being and according to WHO definition refers to ‘an individual's perception of their position in life in the context of the culture and value system in which they live and in relation to goals, expectations, standards and concerns’ [38]. 

It has been shown in the literature that increased burden is significantly related to decreased health–related quality of life among stroke caregivers particularly in mental health and social functioning domains [30, 39]. Several other determinants and predictors of QoL have been reported, such as: physical disability of the stroke survivor, behavioral disturbances following stroke, personal attributes and depression of the caregiver and social support [17, 33, 40]. They are more or less similar to the predictors of the sense of burden, and their role in the quality of life as in burden may vary between the acute and chronic phases of stroke [30].  

In summary, stroke has a great impact on the quality of life of patients and their family careres who provide long–term day–to–day care. Not only the patients but also their caregivers need professional attention and support in order to maintain their own physical and emotional health and well–being. 
